# Optimized clinical practice for superaged patients with hip fracture: significance of damage control and enhanced recovery program

**DOI:** 10.1186/s41038-019-0159-y

**Published:** 2019-08-08

**Authors:** Zaiyang Liu, Jun Zhang, Kaiqi He, Yumei Zhang, Yuan Zhang

**Affiliations:** Department of Orthopedics, Xinqiao Hospital, Army Medical University, Chongqing, 400037 China

**Keywords:** Hip fracture, Superaged patient, Femoral neck fracture, Femoral intertrochanteric fracture, Damage control, Elderly orthopedic care

## Abstract

With the advent of global aging, the incidence, mortality, and medical costs of hip fracture among aged patients are increasing annually. The number of controlled clinical studies and health economics analyses that conform to evidence-based medicine principles is growing day by day. However, unfortunately, no specific recommendations regarding the procedures for the treatment of hip fracture are available. Meanwhile, the existence of both traditional treatment systems and new treatment theories means that most doctors confront difficult choices in their daily practice. These factors make the therapeutic approach for aged patients, especially among superaged patients with hip fracture, extremely challenging. This study focuses on superaged patients (> 80 years as defined by the World Health Organization) with hip fracture and includes their preoperative pathological condition; therapeutic decision-making in terms of the benefit and risk ratio, damage control theory, and enhanced recovery after surgery were also investigated. These patients were discussed specifically by combining the current treatment strategies from several experts and the results of a meta-analysis published recently. The study presents some new ideas and approaches currently recognized in the field, such as preoperative assessment, surgical planning, safety consideration, complication intervention, and enhanced recovery implementation, and further presents some clear interpretations regarding misunderstandings in clinical practice. Finally, optimized treatment according to damage control principles and enhanced recovery after surgery during the perioperative period among superaged hip fracture patients is defined.

## Background

**Fig. 1:**
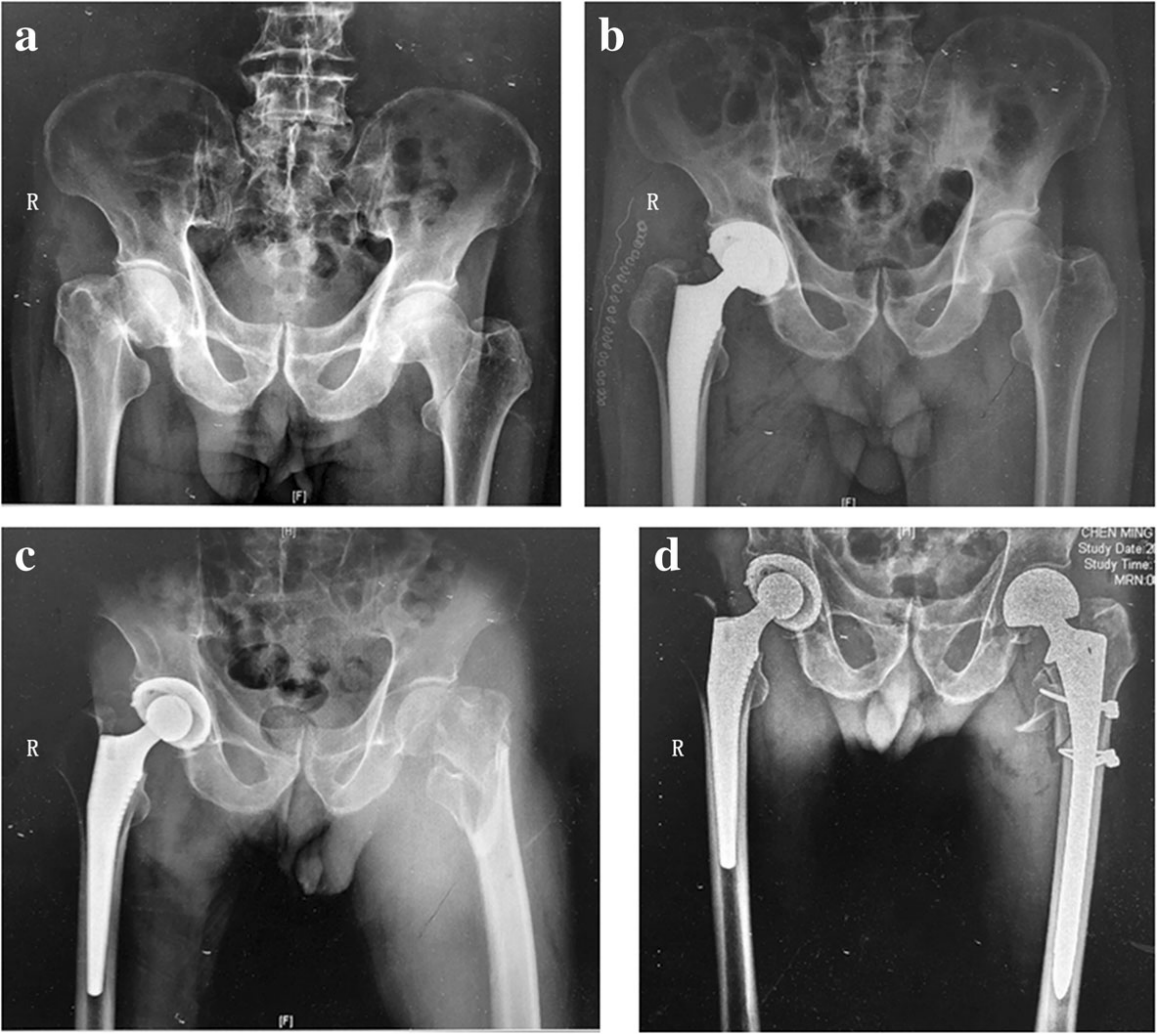
An 81-year-old male patient with sequential hip fractures. **a** Antero-posterior pelvic X-ray showing femoral neck fracture in the right hip upon admission to the hospital. **b** The patient was treated in an optimized practice for superaged patients with hip fracture, including total hip replacement within 48 hours after injury. **c** A fall 1 month later resulted in a femoral intertrochanteric fracture of the left hip. **d** The patient was managed with damage control principles and enhanced recovery after surgery, including femoral head replacement within 48 hours after injury

Hip fracture in aged patients is a common type of osteoporotic fracture, which is common in females over 65 years old and males over 70 years old. Epidemiological analysis revealed that over 1.6 million new cases were found in 2017, and the annual growth is anticipated to be 25%. Therefore, there will be a total of 6.3 million cases in 2050 [1]. The mechanism of hip fracture in aged patients is low-energy trauma (falls, slips, etc.). Approximately 47% of hip fractures are identified as subcapital femoral neck fractures, and 38% are located in the intertrochanteric region [2]. The prognosis is not optimistic; one in three patients dies within the first year after injury, while survivors have poor quality of life. The recovery rate indicates that less than 15% of patients fully recover to their preinjury level of activity. Another difficult problem is that hip fracture significantly increases the risk of contralateral injury [3]. According to the literature, the risk of contralateral hip fracture varies from approximately 5.5% to 20%. Senile dementia, Parkinson’s disease, respiratory disease, and the absence of osteoporosis medical treatment are the variables associated with a higher risk of contralateral fractures [4–6] (Fig. 1).

In this essay, we reviewed a number of studies about the therapy and recovery of hip fracture in superaged patients, with the addition of experiences in our department, aiming at summarizing and looking for better treatment strategies of hip fracture in superaged patients. Our goal is to give patients the best prognosis under current medical conditions. The treatment of hip fracture in superaged patients is unique and complicated. Even with aggressive medical intervention, many complications occur, and the mortality rate is high. The literature indicates that the mortality rate of these patients is between 2.3 and 13.9% during hospitalization, and even after surgical intervention, the mortality rate is still as high as 12 to 23% 6 weeks after surgery. These rates are 6–15 times higher than the rates for elective hip surgery [7]. Even after hip fracture surgery, many older patients develop disabilities in mobility, and more than 30% do not regain independent ambulation [8]. Fortunately, since 2017, effective health education and fall prevention measures have contributed to the global reduction in the incidence rate for females aged over 85 years [3]. It is worth noting that 75% of patients do not die from the pathology of the hip fracture itself but from underlying disease preinjury, such as chronic obstructive pulmonary disease, coronary heart disease, Alzheimer’s disease, visual and hearing impairment, cancer, diabetes, and hypertension. The impact of hip fracture impairs inner homeostasis, which leads to continuous deterioration in the patients’ general condition.

Therefore, the treatment for hip fracture in aged patients should focus on the recovery of homeostasis in their general condition, while treatment of the hip fracture itself is not often a priority. With an understanding of this particular disorder, an increasing number of experts advise deviating from the traditional approach to trauma treatment and include extra-traumatology factors in practice, such as in geriatric orthopedics. This trend has made a significant difference in the treatment of elderly and young patients with hip fracture [9].

## Review

### Risk assessment and preoperative intervention

Management options for hip fractures in superaged patients are always determined by the risk-benefit ratio. Medical treatment will be ethically justified only if there is a greater benefit or a superior result. However, currently, the risk-benefit ratio is difficult to quantify, despite some scoring systems that can assess perioperative complications and mortality by integrating the patients’ demographic characteristics, risk factors, disease susceptibility, and admission test results, from a relatively concise form such as the American Society of Anesthesiologists (ASA) score, the Nottingham score, and the Physiological and Operative Severity Score for the enUmeration of Mortality and Morbidity (P-POSSUM) score, into certain complex scales, such as the Charlson comorbidity index, the cumulative illness rating scale, and the geriatric index of comorbidity. Common problems are the limitations in feasibility, accuracy, and operability [10].

Approximately 70% of elderly patients with hip fractures also have underlying disorders of the respiratory, neurovascular, psychological, cardiovascular, and endocrine systems. This complexity means the doctor must spend more time evaluating these nonspecific pathologies and excluding their impact on the surgical plan [11]. The patients always have to rotate through several departments, including the emergency, traumatology, orthopedics, geriatrics (often separated into several other departments in China), and anesthesiology departments. The stress of the trauma magnifies these initial pathological changes leading to severe complications in a cascading manner. For most conditions, emergency treatment during the golden hour is unattainable.

Careful preoperative examination should not compromise the golden intervention time in aged patients with hip fracture. However, currently, implementation requires agreement among surgeons, anesthesiologists, and geriatricians, and credible communication with the patient’s family. A careful preoperative examination is not equivalent to a complete system examination. For example, 30% of patients over 70 years old have comorbid coronary heart disease, and 60% have comorbid hypertension; if all of them require system examination, such as coronary angiogram, dynamic electrocardiography, and ambulatory blood pressure monitoring, the incidence of acute and long-term complications will increase quickly, and the risk-benefit ratio will multiply exponentially. Some case-control studies indicate that the mortality rate increased by 19.7% when the operation was delayed by 48 h based on cardiac test results [12]. Hence, some experts advocate early treatment without ultrasonic cardiograms, except for acute coronary syndrome with elevated ST segments [13].

Data from evidence-based medicine has confirmed that surgical treatment is the primary option for superaged patients with hip fractures. The complication and mortality rates for nonoperative patients were 6–8 times higher than for operative patients in the first 6 months after injury, regardless of the patients’ surgical tolerance. In other words, there is no other effective treatment that can provide the long-term benefits of surgery. A randomized controlled trial of 191,873 cases and 35 studies showed that mortality can be significantly reduced by early surgical treatment (within 24–48 h) (relative ratios (RR) = 0.74, 95% confidence interval (CI) = 0.67–0.81) [14]. The surgical plan is the essence of preoperative planning; it also determines the prognosis of superaged patients with hip fracture. From a cost-effectiveness and health economics perspective, both emergency surgery and delayed surgery have their advantages and disadvantages [15]. However, most experts agree that the risk-benefit ratio is lowest when the surgery is performed within 24–48 h after injury (mid-level recommendation) [16]. As a result, the international guidelines suggest emergency or nonelective surgery for hip fracture among the aged population, even though 47 to 60% of the surgeries were performed 48 h after injury all over the world. It is estimated that 51% of the delays in surgery were due to organization and transportation reasons; 44% were due to clinical reasons, such as preoperative assessment and treating underlying disease; and 2% were due to anesthesia [17].

In contrast, some experts hold different views; they believe that the underlying diseases in these aged patients with hip fracture experience a “second strike” by hip trauma. Therefore, the treatment for these patients should follow the general principle of damage control. A retrospective cohort study compared the early clinical results among femoral neck fracture patients with chronic renal failure. The surgical intervention was performed 3 to 10 days after injury according to a chronic kidney disease functional score. The results show that delaying the surgery did not reduce the early clinical effects of total hip replacement [18]. The objective is the reduction in actual complications and mortality to levels below the theoretically predicted levels by using the damage control strategy. Furthermore, the authors proposed that the choice of treatment for aged patients with hip fractures be based on damage control theory in their later papers [19]. If the assessment score is classified as low (ASA-PS < 20%, P-POSSUM < 60%), aggressive surgery is advised. If the score exceeds the low-risk level, various interventions need to be performed to stabilize the situation, followed by a new round of scoring.

For some currently widely employed preoperative measures, studies derived from evidence-based approaches have assessed their value to the overall outcome. Interestingly, there is no strong recommendation for preoperative skin traction of the lower extremity because limited evidence suggests that patients benefit from pain relief or fracture reduction (level B evidence). Instead, oxygen therapy (level B recommended), preemptive analgesia (emergent femoral nerve block, level A recommended), and bedsore prevention (level A recommended) are considered significant [20, 21].

The effect of basic medication on surgery is controversial at present, since a large portion of patients are administered antithrombin and antiplatelet agents. Aspirin and clopidogrel are most commonly used. For single use, surgery at 12 h after withdrawal would not increase the risk of bleeding due to their short half-life and high plasma clearance; however, the conclusions from the literature are quite equivocal on the combined use of aspirin and clopidogrel. Therefore, the author still suggests that withdrawal should allow enough time before surgery (level C evidence, usually 3–5 days) [22]. For patients with an International Normalized Ratio (INR) > 1.5 who take warfarin, vitamin K is generally not beneficial for rapid correction of INR, and aggressive intervention with prothrombin complex concentrates is recommended at a dose of 20 IU/kg [23]. More attention should be paid to novel oral anticoagulants, such as dabigatran, apixaban, rivaroxaban, and ticagrelor. Because of their strong inhibition of coagulation function, their half-life of over 12 h, and their lack of specific antagonists, time since withdrawal of as many as five times the half-life of the anticoagulant is considered to be safe for surgery [24]. Other medications that may affect the stability of blood pressure intraoperatively, such as reserpine, would seriously affect catecholamine’s effect on sympathetic excitability. Selegiline, a monoamine oxidase inhibitor, induces an intraoperative hypertensive crisis. Both may increase the risk of anesthesia accidents and cardiovascular risk. Therefore, it is generally recommended that these medications be ceased at least 1–2 weeks before surgery. Other medications, such as β-blockers, statins, and benzodiazepines, have been proven to have no obvious effect on surgery.

### Surgical plan and safety considerations

This paper does not focus on the selection of anesthesia methods. Most scholars have agreed that regional blocks, such as spinal anesthesia, are better than general anesthesia regarding postoperative benefit. A meta-analysis involving 18,158 patients found that regional anesthesia resulted in lower in-hospital mortality and fewer pulmonary complications [25]. However, intraspinal anesthesia may be contraindicated for patients currently using anticoagulants. Hemodynamic optimization has been advocated for the surgery of superaged patients with hip fracture in recent years. In addition, targeted nerve blocks provide a new approach to enhance recovery after surgery; they include obturator nerve and lateral femoral cutaneous nerve blocks, which will be beneficial for pain relief [26], and iliotibial membrane block, which will help reduce the incidence of postoperative delirium [27].

There is still debate about osteosynthesis or arthroplasty as surgical options among doctors specializing in joint reconstruction and trauma. Although the surgical plan will be affected by the fracture site and type, the current consensus is that a personalized plan should be developed based on the patient’s overall condition, mental and cognitive state, social functioning, independent living ability, local bone quality, etc. In that regard, arthroplasty is undoubtedly the priority to achieve early weight-bearing and functional training for superaged patients, especially for patients with cognitive dysfunction and respiratory diseases. A large sample cohort study revealed that the reoperation rate 1 year after surgery was extremely high in the osteosynthesis group (23.7%) compared to 3.4% in the arthroplasty group and revealed higher pain scores and patient dissatisfaction rates [28].

**Fig. 2:**
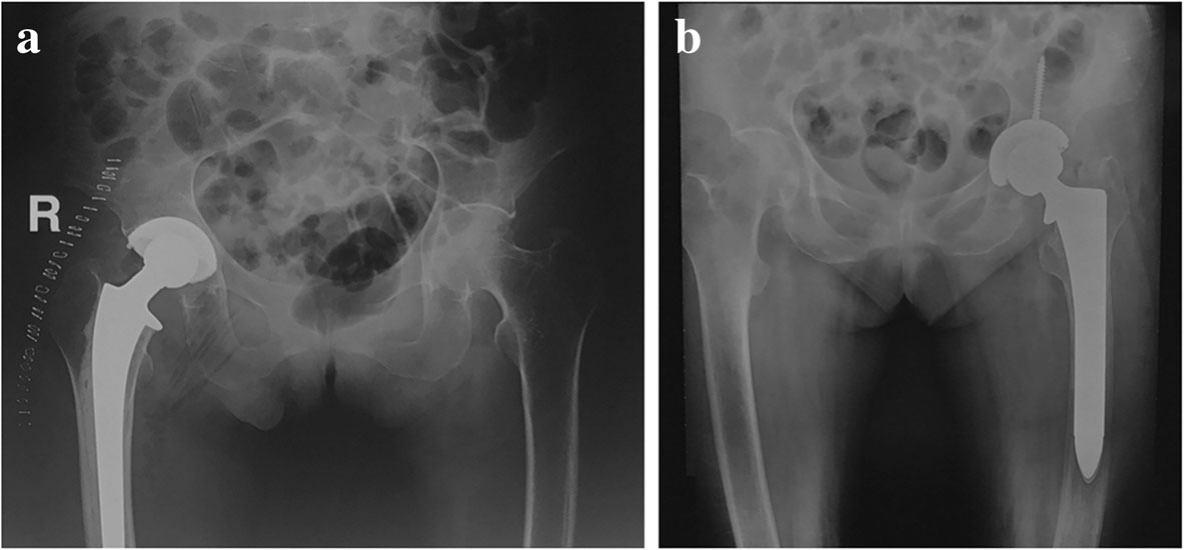
Proximal cortical bone resorption and medullary cavity expansion, known as Dorr C morphology, are usually identified in superaged patients with hip fractures. Evidence-based medicine showed **a** cemented prostheses had better surgical safety and clinical outcomes than **b** cementless prostheses

The choice of prosthesis for arthroplasty in superaged patients is usually difficult and requires special considerations. Cemented prostheses seem to be advantageous for load transfer, reoperation rate, and pain score, while the risk is for fat embolization and bone cement toxicity. Therefore, the author recommends specific techniques, including decompression of the medullary cavity, cement mixing in a vacuum, and optimized cement implantation. A single-blind controlled study from the Swedish joint registration system reported the 2-year follow-up results from 69 cases of displaced femoral neck fractures. Using cemented and cementless femoral prostheses, they found a notable increase in periprosthetic fracture and dislocation using cementless prostheses (22.8% higher than that of the cemented group) [29]. The authors believe that the advantages of cemented prostheses in superaged patients with hip fracture are (1) better femoral medullary cavity compliance and mechanical stress transfer, (2) ideal penetration and cross-linking of the bone cement, and (3) a lower incidence of intraoperative occult fractures (Fig. 2).

In addition, compared with total hip arthroplasty (THA), femoral head replacement has limited advantages regarding surgical time and intraoperative hemorrhage, while there is no evidence for better short-term or long-term results. For patients with femoral neck fractures below the femoral head, THA has a better long-term result than femoral head replacement [30]. However, many studies in the last 3 years have shown that femoral head replacement using a direct anterior approach (DAA) can provide higher postoperative satisfaction and improved survival rates and functional scores for older patients [31]. Dual mobility and a restrictive acetabular prosthesis are needed in patients with weak abductors caused by Parkinson’s disease, cerebral thrombosis, or the sequelae of hypoxia [32].

Minimally invasive surgery has been proven to have potential advantages in reducing surgical trauma, bleeding, and the risk of blood transfusion. For exposure, the conventional posterolateral approach requires an incision of 10–15 cm, cut from the external rotator muscle of the hip and the trochanteric branch of the lateral femoral artery. In addition, there are defects such as considerable blood loss, a high blood transfusion rate, possible sciatic nerve injury, severe postoperative pain, and a long period of postoperative position limitations. Therefore, the DAA is advocated by an increasing number of hip doctors in Europe and America. In the USA, 40 to 50% of doctors have switched their surgical approach from the posterior lateral to the DAA. This approach involves entering through the neuromuscular space between the rectus femoris/tensor fascia lata and the femoral nerve/superior gluteal nerve. It is currently the only minimally invasive approach among all current THAs.

It has the advantages of a small incision (6–8 cm), a short operation time, no interference with the external rotator muscle, reconstruction of the joint capsule, accurate positioning of the prosthesis, slight postoperative pain, and early unlimited functional activity [33]. Therefore, DAA is considered to be the most effective way to enhance recovery after surgery in hip arthroplasty both in theory and in clinical practice. However, DAA has a steep learning curve and a high risk of early intraoperative complications. Surgeons need standardized training, help from experienced surgeons, and experience with 50–100 cases for learning before reaching a stable plateau. In addition, beware of technical pitfalls when treating patients with comminuted intertrochanteric fractures (EVAN III–IV), serious osteoporosis, and rheumatoid arthritis.

Regardless of the surgical approach, surgical trauma control should be the primary concern. Theoretically, the surgeon should use the smallest incision, cut open limited tissue by using limited or minimally invasive tissue release techniques, and use temporary reduction and stabilization of the fracture block and implant and soft tissue reconstruction techniques to complete the operation.

### Postoperative intervention and enhanced recovery after surgery

**Fig. 3:**
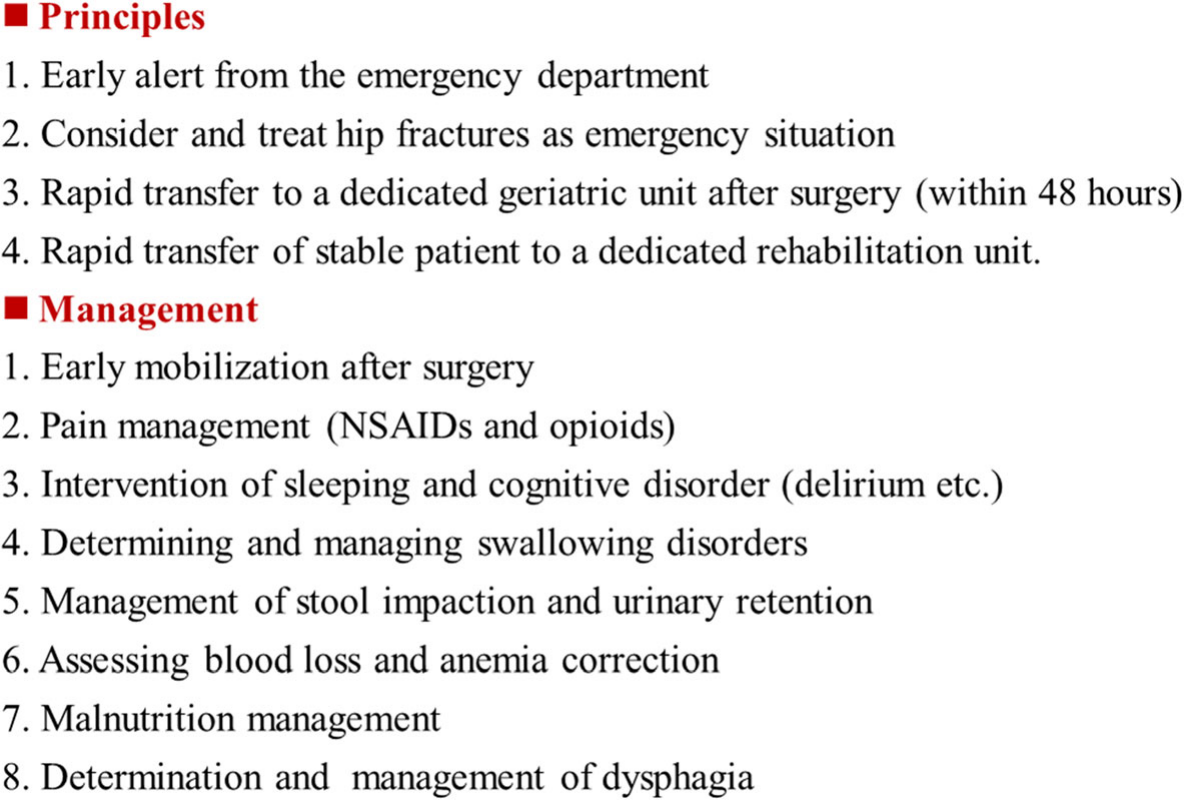
Principles and managements of superaged patients with hip fracture in an orthogeriatric care model mode. *NSAIDs* nonsteroidal antiinflammatory drugs

Perioperative rehabilitation of superaged patients with hip fractures is as important as the surgery itself. Because of this unique pathological state, European and American doctors proposed the concept of the orthogeriatric care model (OCM). The core of the concept involves transferring patients immediately after the operation to a special ward, which is similar to the environment before injury, where the treatment is performed by geriatric orthopedic doctors. OCM can significantly reduce mortality in the first 6 months after surgery (odds ratio (OR) 0.43, 95% CI 0.25–0.73). In addition, the incidence of postoperative bedsores, the readmission rate within 30 days, and the Intensive Care Unit rate decreased by 50% [34]. Geriatric syndrome is the focal target of OCM, which includes delirium, dysphagia, deep vein thrombosis of the lower limb, anemia, and malnutrition (Fig. 3).

The authors believed that the recent increase in multidisciplinary treatment (MDT) for aged patients with hip fractures is actually an attempt to implement OCM. Although enhanced recovery after surgery (ERAS) is the specific focus of OCM, our department has been working on this for more than 3 years; we would like to elaborate our procedures as follows.

#### Systemic inflammation

The process of aging is accompanied by chronic and low-level systemic inflammation. Once hip fracture occurs, the trauma leads to intracellular mitochondrial fragmentation and induces the release of some proinflammatory factors similar to bacterial infection, such as tumor necrosis factor (TNF)-α, interleukin (IL)-6, and IL-1. Experimental results from animal models revealed that the first attack of systemic inflammation is usually targeted to acute lung injury. Studies of molecular pathways suggest that this result is mediated by upregulated expression of toll-like receptor 9 (TLR9)/nuclear factor kappa B (NF-kB) in broken mitochondria. The high expression of the latter two molecules will persist until 6 weeks after surgery [35]. This is consistent with the clinical observation that plasma D-dimer and C-reactive protein (CRP) return to the normal range, while complications and mortality become stable at 6 weeks after surgery [36]. Therefore, we advocate that proper suppression of systemic inflammation in superaged patients with hip fracture is necessary. Our clinical work also showed that low-dose short-acting corticosteroids (100 mg hydrocortisone or 40 mg prednisone) have positive effects in terms of stimulating the stress response of the pituitary-adrenal gland axis, decreasing ischemia-reperfusion injury, stabilizing the vital signs on the day after surgery, and reducing the incidence of delirium and infection during hospitalization. Basic research and clinical observation have also shown that the plasminogen inhibitor tranexamic acid has anti-inflammatory effects and can reduce perioperative blood loss and the blood transfusion rate through the mechanism of reducing traumatic stress [37]. Therefore, an expert consensus on total hip and knee arthroplasty in China regarding the perioperative sequential dosage regimen of anticoagulants has proposed a sequential dosage regimen of tranexamic acid before, during, and after surgery [38].

#### Delirium

Dementia is one of the main causes of high mortality in superaged patients with hip fracture. Studies have shown that the occurrence of dementia in this population was 34.3% when patients were admitted to a hospital, and it could reach 57.7% after surgery [39]. Postoperative delirium is usually indicated by early cognitive impairment, such as memory loss, mental disorders, distraction, and even an alteration in consciousness, which seriously affects the postoperative recovery of patients. Some related risk factors include being superaged, having preoperative cognitive impairment or depression, having used psychiatric drugs (mental drug, antiemetic drug, benzodiazepines), and having hydroelectrolyte imbalance, audio-visual impairment, chronic pain, urinary retention, or constipation. Studies have shown that decreased plasma dehydrodione and rostenedione and increased cortisol levels can be used to evaluate the risk of postoperative delirium [40]. Preventive measures targeting these risk factors, such as oxygen therapy, hydroelectrolyte balancing, sufficient analgesia, adequate nutrition, dynamic monitoring of mental state, and timely transfer to the geriatric ward, are expected to reduce the occurrence of postoperative delirium, dislocation, fracture, and other complications [41]. For patients with clinical symptoms, oral or intramuscular administration of haloperidol is considered to be effective, but intravenous injection is not recommended due to the incidence of arrhythmia. Additionally, long-term use of haloperidol requires monitoring of the ECG QT interval (< 450 ms or < 25% basic value). In addition, there is a misunderstanding with regard to treating postoperative delirium; most doctors limit their use of opioids because of concerns about the inhibitory side effects. In fact, most postoperative delirium is caused by inadequate analgesia, so the benefits of opioid use after surgery are far greater than the risks [42].

#### Systemic and local metabolic disorders

Malnutrition is a common state in elderly patients before injury. This state, which is aggravated by the trauma event, further increases therapy costs and hospital stays and induces acute geriatric syndrome, especially delirium. Existing laboratory tests (such as hemoglobin and plasma albumin/proalbumin) and nutrition assessment scales (such as ICD-10-AM) are not efficient enough for assessing nutritional status [43]. There is a study in hip fracture patients that shows metabolic syndrome is independently associated with increased odds of any adverse event and increased aggregate morbidity [44]. Therefore, preoperative energy supply (e.g., oral short peptides 4 h before surgery and a clear fluid diet 2 h before surgery) is vital to compensate for the intraoperative energy loss. Furthermore, intensive nutrition supplementation (full protein nutrition formula) for 1–2 weeks after surgery was shown to be significant in reducing the incidence of postoperative complications. For example, clinical data from our department from 2016 to 2017 showed that the rate of wound healing problems, superficial infection, and periprosthetic joint infection were decreased by 50 to 70%. The European Society of Parenteral Enteral Nutrition (ESPEN) emphasizes that oral nutrition supplements should be extended to 2 months after surgery among elderly patients [45]. However, one factor limiting implementation is the lack of patient compliance caused by the expense and change in living habits.

Metabolic disorders also occur in the bone niche, and one significant challenge in superaged patients is the imbalance in synthesis and degradation of collagen/minerals, as well as dysfunction of osteoblasts/osteoclasts in the bone microenvironment. Stimulation of osteogenic function, inhibition of osteoclast activity, and enhancement of local bone density and mineralization capacity are proven to be effective in the primary prevention of hip fracture in the elderly population. Thus, the need for long-term anti-osteoporosis treatment in elderly patients with hip fracture is urgent. Specific anti-osteoporosis programs should be developed according to the individuals’ bone quality. Medication with calcium, vitamin D, calcitonin, bisphosphonates, parathyroid hormone, and estrogen receptor antagonists, combined with lifestyle adjustment and physical therapy, is expected to play a major role in enhancing the stability and extending the survival life of the prosthesis and preventing secondary fracture and the need for revision surgery. Patients whose Norton score is < 14 should be informed of the high risk of multiple falls and refracture surgery, and adequate health education and medical interventions should be emphasized [46].

#### Dysphagia

Dysphagia might be a neglected complication in current clinical work. Studies have shown that up to 34% of patients without dysphagia will develop oropharyngeal dysphagia within 72 h after THA [47]. Preoperative neurologic or respiratory diseases, as well as cognitive impairment, may be the main causes. Dysphagia may lead to postoperative hydroelectrolyte disorders, nutritional imbalances, aspiration pneumonia, and increased constipation, which in turn can lengthen hospitalizations and increase costs. Currently, it is difficult to manage postoperative dysphagia, and medical treatments (such as gastrodynamic drugs) often result in fair outcomes. Posture adjustment, swallowing training, and diet optimization are the most commonly recommended treatments at present [48]. Clinical observation in our department showed that dysphagia was related to several preoperative factors, among which the most common ones were time spent fasting and sleep quality. The incidence of dysphagia was 2–3 times higher in patients who fasted beginning at 10:00 p.m. the night before surgery compared to those who fasted for 4 h and stopped drinking for 2 h before surgery. Sleep management the night before the operation (zolpidem) and early postoperative swallowing training (such as drinking adequate water) can effectively prevent the incidence of dysphagia.

#### Anemia

Anemia in elderly patients with hip fractures is a common sign of deterioration in hematopoietic function. Epidemiological data have confirmed that 80% of elderly patients with hip fractures were found to be anemic (hemoglobin below 11 g/L) when first admitted to the hospital [49]. Records from our data, including 87 patients over 80 years old who were in our department from 2015 to 2017, demonstrated that the proportion of patients whose hemoglobin was lower than 9 g/L was as high as 87.2%; if dehydration after injury were taken into account, the ratio would be higher. However, anemia is usually asymptomatic or subclinical before surgery, and it can lead to dysfunction in multiple systems after traumatic stress, further increasing infection rates and hospital stays and decreasing quality of life. If intraoperative hemostatic measures are insufficient or if they are not compensated by blood transfusion, acute complications and mortality will be significantly increased. Correspondingly, correction of anemia is considered one important part of perioperative blood management to enhance recovery after total hip and knee arthroplasty in China according to expert consensus; they also advocate actively correcting primary hemorrhagic diseases and providing nutrition guidance, a balanced diet, and medical intervention [50]. If there is no contraindication, one dose of erythropoietin (EPO, 4000 IU) was given 1 week preoperatively, 1 day after admission, and 1 day postoperatively, and with iron therapy, EPO can stimulate red blood cell mobilization, improve the oxygen carrying capacity of hemoglobin, and promote early ambulation. As mentioned previously, tranexamic acid can also reverse postoperative anemia by reducing red blood cell loss in interstitial tissue and inhibiting pericapillary inflammation [51].

#### Thrombosis

A cohort study of a Korean population showed that the incidence of deep venous thrombosis (DVT) in elderly patients was 2.6% within 24 h of hip fracture, and it increased to 13.3% if the injury was delayed to 72 h [52]. Even with standard anticoagulant measures, the incidence of perioperative DVT in elderly patients with hip fracture was 6–8 times higher than that in patients with selective THA [53]. However, there is no significant difference in the incidence of postoperative mortality due to acute pulmonary embolism. Therefore, for superaged patients with hip fracture, coagulation function tests and thrombus risk assessments should be performed immediately after admission. After weighing the risk of thrombus/hemorrhage, sufficient anticoagulant therapy (e.g., low molecular weight heparin, rivaroxaban, and aspirin) should be given as soon as possible according to the guide for the prevention of venous thrombosis in orthopedic operations in China. For patients with a higher risk of hemorrhage, mechanical prophylactic measures (e.g., use of a sole vein pump, intermittent use of pneumatic compression devices, or use of gradual compression stockings) are highly recommended (class IA). For patients with an extremely high risk of thrombosis (such as European System for Cardiac Operative Risk Evaluation (Euro SCORE) > 6 and CHADS2 score (congestive heart failure, hypertension, age > 75 years, diabetes mellitus, prior stroke and TIA) > 2), studies show that the anticoagulant period should be extended or the anticoagulant intensity should be increased according to the recommendations of the specialist [54].

## Conclusions

The complexity of perioperative management in superaged patients with hip fracture lies in the workflow for managing the combination of underlying diseases, and it has been the key factor in decision-making for surgical treatment and postoperative recovery. The principles of damage control surgery should be beneficial in this area as described above. Definitive surgery was often performed after stabilization of the patient’s internal condition by minimizing secondary effects, coagulation disorders, and hydroelectrolyte imbalance. At present, it is urgently important to maximally reduce the time consumed by comprehensive preoperative examination and to optimize the transport procedure after admission. Patients who conform to the principle of emergent surgery usually represent a relatively small proportion with comprehensive risk assessment scores at a low level. Enhanced recovery after surgery can be fully performed in these patients. However, evidence-based medicine proves that the timing of surgery is not the only factor that determines the patient’s postoperative morbidity and mortality. We cannot measure the quality of treatment in terms of emergent surgery or delayed surgery. Elective surgery should not be interpreted as delayed surgery. Elective surgery may be a more appropriate option for the superaged population, but the time window should not be later than 3–5 days after injury. In addition, more communication between doctors and patients is needed. In the end, the comprehensive abilities of the MDT team have proven to be a touchstone for determining early and long-term clinical results.

## Abbreviations

ASA: American Society of Anesthesiologists
CHADS2: Congestive heart failure, hypertension, age > 75 years, diabetes mellitus, prior stroke, and TIA
CRP: C-reactive protein
DAA: Direct anterior approach
DVT: Deep venous thrombosis
EPO: Erythropoietin
ERAS: Enhanced recovery after surgery
Euro SCORE: European System for Cardiac Operative Risk Evaluation
INR: International Normalized Ratio
MDT: Multidisciplinary treatment
OCM: Orthogeriatric care model
P-POSSUM: The Physiological and Operative Severity Score for the enUmeration of Mortality and Morbidity
THA: Total hip arthroplasty
